# Supplementary material for: Language outcomes of preschool children who are HIV-exposed uninfected: An analysis of a South African cohort
Source: PLoS One. 2024 Apr 10;19(4):e0297471. doi: 10.1371/journal.pone.0297471 (PMC11006185; doi:10.1371/journal.pone.0297471)

S1 Figure: Directed Acyclic Graph displaying plausible pathways for the association between HIV exposure and neurocognitive development

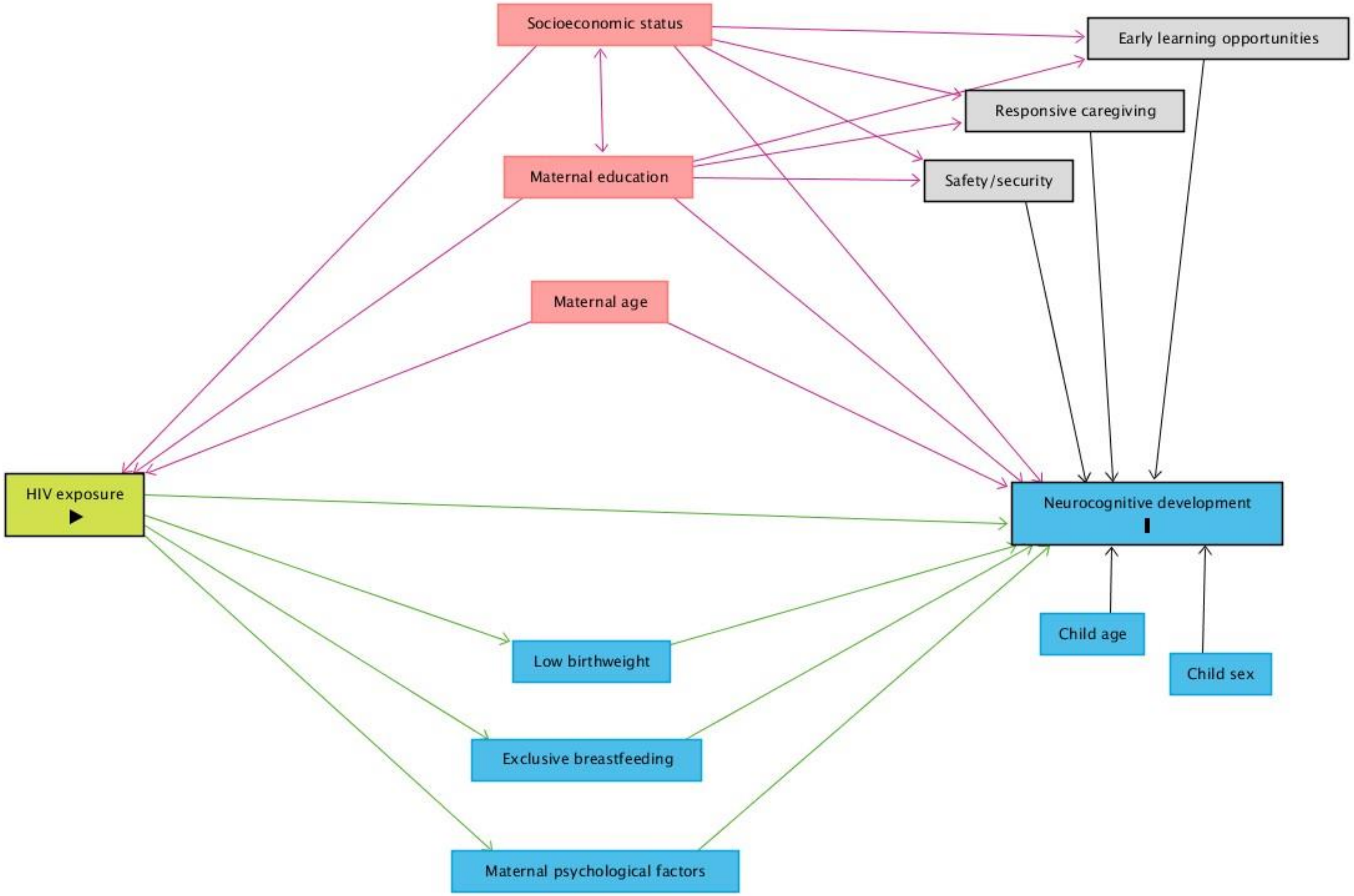

Supplement: S1 Fig — (PDF) [file pone.0297471.s001.pdf]
