# Supplementary material for: Language outcomes of preschool children who are HIV-exposed uninfected: An analysis of a South African cohort
Source: PLoS One. 2024 Apr 10;19(4):e0297471. doi: 10.1371/journal.pone.0297471 (PMC11006185; doi:10.1371/journal.pone.0297471)
Supplement: S1 Table — (PDF) [file pone.0297471.s002.pdf]

S1 Table: Outcome measures

Characteristic	Measure	Details
**General cognitive function**	Kaufman Assessment Battery for Children (KABC-II) - Non-verbal index	The Non-verbal index permits a valid assessment of children who are hearing impaired, have limited English proficiency or have moderate to severe speech or language impairments.
Problem solving	KABC-II Conceptual thinking	The child views a set of 4 or 5 pictures and identifies the one picture that does not belong with the other. Some items present meaningful stimuli, and others use abstract stimuli.
Visual-spatial processing	KABC-II Face recognition	The child attends closely to photographs of one or two faces that are exposed briefly and then selects the correct face or faces, shown in a different pose, from a group photograph.
Visual-spatial problem solving	KABC-II Triangles	For most items, the child assembles several identical foam triangles (blue on one side, yellow on the other) to match a picture of an abstract design; for easier items, the child assembles a set of colourful plastic shapes to match a model constructed by the examiner or shown on an easel.
Working memory/motor sequencing	KABC-II Hand movements	The child copies the examiner's precise sequence of taps on the table with the fist, palm, or side of the hand
**Language**	KABC-II Expressive Language	The child provides the name of a pictured object presented by the examiner
**Memory**	KABC-II Atlantis	The examiner teaches the child the nonsense names for fanciful pictures of fish, plants and shells; the child demonstrates learning by pointing to each picture (out of an array of pictures) when it is named.
