# Supplementary material for: Language outcomes of preschool children who are HIV-exposed uninfected: An analysis of a South African cohort
Source: PLoS One. 2024 Apr 10;19(4):e0297471. doi: 10.1371/journal.pone.0297471 (PMC11006185; doi:10.1371/journal.pone.0297471)
Supplement: S2 Table — (PDF) [file pone.0297471.s003.pdf]

S2 Table: Maternal, child and household characteristics of the full cohort, by HIV exposure

Variable	Full Cohort (n=1141)	CHEU (n=247)	CHUU (n=894)	P-value
Child sex (female)	553 (48.5)	109 (44.1)	444 (49.7)	0.123
Low Birthweight	176 (15.4)	35 (14.2)	141 (15.8)	0.537
Premature Birth	188 (16.5)	47 (19.0)	141 (15.8)	0.474
Maternal age (yrs)	26.9 (5.68)	29.9 (5.25)	26.1 (5.51)	<0.001
Maternal education				
Primary	85 (7.5)	26 (10.5)	59 (6.6)	<0.001
Some Secondary	609 (53.4)	79 (62.8)	454 (50.8)	
Completed Secondary	374 (32.8)	27 (24.29)	314 (35.1)	
Any Tertiary	73 (6.4)	67 (7.5)	32 (7.5)	
Smoker status				
Non-smoker	261 (22.9)	57 (23.1)	204 (22.8)	0.124
Passive smoker	479 (42.0)	118 (47.8)	361 (40.4)	
Active smoker	351 (30.8)	63 (25.5)	288 (32.2)	
Employed	307 (26.9)	61 (24.7)	246 (27.5)	0.376
Exclusive Breastfeeding for 5 months	167 (14.6)	36 (14.6)	93 (17.7)	0.569
Household income				
<R1000/m	431 (37.8)	96 (38.9)	335 (37.5)	0.411
R1000-R5000/m	556 (48.7)	124 (50.2)	432 (48.2)	
>R5000/m	154 (13.5)	27 (10.9)	127 (14.2)	

Footnote. N(%) for categorical variables, mean (SD) for continuous variables. P-values generated using, chi-squared test for categorical variables and t-test for continuous variables. Percentages calculated out of all data. Missing data: prematurity n=5, breastfeeding n=77, alcohol use n=146, EPDS n=50, smoking n=50, water n=5, toilet n=2. Abbreviations: CHEU: Children who are HIV-exposed uninfected, CHUU: Children who are HIV-unexposed uninfected
