# Supplementary material for: Language outcomes of preschool children who are HIV-exposed uninfected: An analysis of a South African cohort
Source: PLoS One. 2024 Apr 10;19(4):e0297471. doi: 10.1371/journal.pone.0297471 (PMC11006185; doi:10.1371/journal.pone.0297471)
Supplement: S3 Table — (PDF) [file pone.0297471.s004.pdf]

S3 Table: Neurocognitive outcomes at 3.5 years compared between CHEU and CHUU in the analytic (complete-case) cohort

Domain	Scores Mean (SD)	Unadjusted mean difference (95% CI)	p-value	Effect size	Adjusted† mean difference (95% CI)	p-value	Effect size
**Cognitive function (score)**							
CHUU	76.55 (14.77)	Reference	0.637	-	Reference	0.672	-
CHEU	75.77 (13.85)	-0.78 (-4.02, 2.47)		-0.05 (-0.28, 0.17)	-0.72 (-4.06, 2.62)		-0.04 (-0.27, 0.17)
**Expressive language**							
CHUU	7.23 (2.03)	Reference	0.026*	-	Reference	0.049*	-
CHEU	6.73 (1.79)	-0.50 (-0.94, -0.06)		-0.25 (-0.47, -0.03)	-0.46 (-0.91, -0.02)		-0.23 (-0.45, -0.01)
**Memory**							
CHUU	7.83 (1.90)	Reference	0.257	-	Reference	0.184	-
CHEU	8.06 (1.45)	0.23 (-0.17, 0.64)		0.13 (-0.09, 0.35)	0.29 (-0.14, 0.71)		0.16 (-0.06, 0.38)
**Domain**	**Sub-optimal development**	**Unadjusted odds**	**p-value**		**Adjusted† odds ratio**	**p-value**	
Cognitive function							
CHUU	254 (63.5)	1	0.518	-	1	0.474	-
CHEU	65 (67.0)	1.16 (0.73, 1.87)		-	1.20 (0.73, 1.99)		-

Footnote: †Adjusting for child sex, maternal education, maternal age and household income. Effect size measured using Cohen's d. *p<0.05.
Abbreviation: CHUU: Children who are HIV-unexposed uninfected; CHEU: Children who are HIV-exposed uninfected.
