# Supplementary material for: Language outcomes of preschool children who are HIV-exposed uninfected: An analysis of a South African cohort
Source: PLoS One. 2024 Apr 10;19(4):e0297471. doi: 10.1371/journal.pone.0297471 (PMC11006185; doi:10.1371/journal.pone.0297471)
Supplement: S4 Table — (PDF) [file pone.0297471.s005.pdf]

S4 Table: Neurocognitive outcomes at 3.5 years compared between CHEU and CHUU among all those completing each assessment

Domain	Scores<br>Mean (SD)	Unadjusted mean difference (95% CI)	p-value	Effect size	Adjusted† mean difference (95% CI)	p-value	Effect size
**Cognitive function (score)**							
CHUU	77.00 (14.84)	Reference	0.687	-	Reference	0.853	-
CHEU	76.41 (14.52)	-0.59 (-3.50, 2.31)		-0.04 (-0.24, 0.16)	-0.28 (-3.25, 2.69)		-0.02 (-0.22, 0.18)
**Expressive language**							
CHUU	7.22 (2.02)	Reference	0.012*	-	Reference	0.038*	-
CHEU	6.69 (1.82)	-0.53 (-0.95, -0.12)		-0.27 (-0.48, -0.06)	-0.45 (-0.88, -0.02)		-0.23 (-0.44, -0.02)
**Memory**							
CHUU	7.89 (1.89)	Reference	0.444	-	Reference	0.261	-
CHEU	8.02 (1.45)	0.13 (-0.20, 0.46)		0.07 (-0.11, 0.26)	0.20 (-0.15, 0.55)		0.11 (-0.07, 0.29)
**Domain**	**Sub-optimal development**	**Unadjusted odds**	**p-value**		**Adjusted† odds ratio**	**p-value**	
Cognitive function							
CHUU	331 (62.57)	1	0.732	-	1	0.756	-
CHEU	79 (64.23)	1.07 (0.71, 1.62)		-	1.07 (0.69, 1.65)		-

Footnote: †Adjusting for child sex, maternal education, maternal age and household income. Effect size measured using Cohen’s d. *p<0.05.
Abbreviation: CHUU: Children who are HIV-unexposed uninfected; CHEU: Children who are HIV-exposed uninfected
